# Prognostic Significance of Ribosome-related Genes Signature in Diffuse Large B Cell Lymphoma

**DOI:** 10.7150/jca.80926

**Published:** 2023-01-22

**Authors:** Wenqi Wu, Jinhuan Wang, Yanan Jiang, Xin Hu, Ye Tian, Long Chen, Huimeng Sun, Yuhang Li, Su Liu, Yangyang Lv, Jing Guo, Hong Xu, Donghui Xing, Yixin Zhai, Linyan Tian, Cheng Li, Xiang He, Kaiping Luo, Yuan Pan, Zhigang Zhao

**Affiliations:** 1 Department of Oncology, Tianjin Medical University Cancer Institute and Hospital, National Clinical Research Center for Cancer, Key Laboratory of Cancer Prevention and Therapy, Tianjin's Clinical Research Center for Cancer, Tianjin, 300060, China; 2 Department of Medical Oncology, Tianjin First Central Hospital, School of Medicine. Nankai University, Tianjin, 300192, China; 3 Department of Senior Ward, Tianjin Medical University Cancer Institute and Hospital, National Clinical Research Center for Cancer, Key Laboratory of Cancer Prevention and Therapy, Tianjin's Clinical Research Center for Cancer, Tianjin, 300060, China; 4 Tianjin Sino-US Diagnostics, Tianjin, 300380, China

**Keywords:** DLBCL, ribosome-related genes, prognostic model, targeting therapy, NLE1

## Abstract

**Background:** The diffuse large B-cell lymphoma (DLBCL) is a heterogeneous lymphoma with a dismal outcome, due to approximately 40% patients will be relapsed or refractory to the standard therapy of rituximab plus cyclophosphamide, doxorubicin, vincristine and prednisone (R-CHOP). Therefore, we need urgently to explore the approach to classify the risk of DLBCL patients accurately and accurately targeting therapy. The ribosome is a vital cellular organelle that is mainly responsible for translation mRNA into protein, moreover, more and more reports revealed that ribosome was associated with cellular proliferation and tumorigenesis. Therefore, our study aimed to construct a prognostic model of DLBCL patients using ribosome-related genes (RibGs).

**Method:** We screened differentially expressed RibGs between healthy donors' B cells and DLBCL patients' malignant B cells in GSE56315 dataset. Next, we performed analyses of univariate Cox regression, the least absolute shrinkage and selection operator (LASSO) regression and multivariate Cox regression analyses to establish the prognostic model consisting of 15 RibGs in GSE10846 training set. Then, we validated the model by a range of analyses including Cox regression, Kaplan-Meier survival, ROC curve, and nomogram in training and validation cohorts.

**Results:** The RibGs model showed a reliably predictive capability. We found the upregulated pathways in high-risk group most associated with innate immune reaction such as interferon response, complement and inflammatory responses. In addition, a nomogram including age, gender, IPI score and risk score was constructed to help explain the prognostic model. We also discovered the high-risk patients were more sensitive to some certain drugs. Finally, knocking out the NLE1 could inhibit the proliferation of DLBCL cell lines.

**Conclusion:** As far as we know, it is the first time to predict the prognosis of DLBCL using the RibGs and give a new sight for DLBCL treatment. Importantly, the RibGs model could be acted as a supplementary to the IPI in classifying the risk of DLBCL patients.

## Introduction

The diffuse large B-cell lymphoma (DLBCL) belonging to non-Hodgkin lymphoma (NHL), is the most common type lymphoma in adult^1^, furthermore the incidence increased markedly in recent years and ranked the top ten common cancers^2^, ^3^. The combination of rituximab, cyclophosphamide, doxorubicin, vincristine, and prednisone (R-CHOP), is the first line treatment for DLBCL patient for several decades^4^. Although 50-70% patients get completely remission (CR)^5^, 15-25% patients are primary refracted to standard treatment and 20-30% of patients will relapse after an initial complete response^6^.

The prognosis of DLBCL is heterogeneous, which is influenced by age, disease stage and gene-expressed profiling. Usually, the International Prognostic Index (IPI) risk score, including stage, age, lactic dehydrogenase (LDH), performance status, and extra-nodal sites involved, is the first choice to assess the risk of patient with DLBCL. Recently, the revised IPI (R-IPI)^7^ and National Comprehensive Cancer Network -IPI (NCCN-IPI)^8^ were established to improve the discrimination of patients treated with R-CHOP. However, the IPI risk score, R-IPI and NCCN-IPI were based on the 5 clinical variables, they are limited in accurately prediction individualized therapy of DLBCL patients.

The gene-expression profiling helps us to predict the prognosis of DLBCL patients. For instance, the activated B-cell-like (ABC) and germinal-center B-cell-like (GCB) subgroups of DLBCL are distinct by expression of CD10, BCL6 and IRF4/MUM1^9^-^11^, and the patients of GCB subgroup have a higher CR ratio than non-GCB^12^. In addition, the patients with rearrangements of MYC and BCL2 and/or BCL6 gene, or double-protein expression are associated with a more aggressive clinical course and poor prognosis^13^. Therefore, making the gene-expression into consideration will help us classify the risk of DLBCL accurately and contribute to target therapy.

The ribosome is the most ancient primordial and crucial molecular machine in mammalian cell, which is responsible for translating mRNA into protein^14^. The assembly and function accurately are very important in ribosomal biogenesis, because a range of diseases are associated with defects in ribosome proteins (RPs), rRNA processing or ribosome assembly factors^15^. Previous reports showed that patients with ribosomal mutations have a higher risk to develop cancer in their life, moreover, in particular cancer the risk even up to 200-fold higher^16^-^18^, and a variety of tumors were discovered somatic mutations in RPs. Accordingly, the tumor cells have mutations in ribosome-associated genes frequently, for example, heterozygous loss of RPL5 occurs in 30% of multiple myeloma, melanoma, glioblastoma and breast cancers^19^-^22^, and 2% of T-ALL patients^23^, ^24^. Furthermore, the oncogenes and loss of suppressor genes will enhance the activities of ribosome biogenesis^25^, ^26^. On the other hand, the ribosome-related genes could be targeted to anti-tumor, as the mammalian target of rapamycin complex1 (mTORC1) regulates the ribosome biogenesis showed a noticeable inhibition of growth in tumor cells^27^. In summary, more and more studies showed that the ribosome plays a key role in tumorigenesis and association with prognosis^28^. Therefore, we chose the RibGs to construct the prognostic model of the DLBCL.

In our study, we constructed a model that calculated the risk score using the mRNA expression of RibGs to predict the prognosis of DLBCL patients pioneeringly, which showed a better predictive capability than present approach in DLBCL prognosis.

## Materials and methods

### Data Acquisition

The 2591 ribosome related genes (**Supplementary Table S1**) were collected from the GSEA database (https://www.gsea-msigdb.org/gsea/index.jsp). The GSE56315 contains the gene-expression data of B cells of normal and DLBCL patients. The clinical and genes expressed information came from these datasets including GSE10846, GSE11318 and GSE87371. The GSE datasets were retrieved from Gene Expression Omnibus (GEO) database (https://www.ncbi.nlm.nih.gov/geo/). The detail information is presented in **Table 1**.

### Differentially Expressed Ribosome Related Genes

There were 2357 ribosome related genes commonly in GSEA and GSE dataset (**Supplementary Table S1**). Differentially expressed genes between normal B cells and DLBCL B cells were analyzed by the limma package in R software with the Wilcoxon test according to Log2Fc < -1 or > 1 and p < 0.005 (**Supplementary Table S1**). The upregulated genes were functional enriched by clusterProfiler package. The top 15 pathways (**supplementary Table S1**) from Gene ontology analysis were showed by bar plots.

### Construction of the Prognostic model

Firstly, the univariate Cox analysis was applied to screen genes related to overall survival (OS) in GSE10846 training set. Secondly, analysis of the least absolute shrinkage and selection operator (LASSO) Cox regression was used to get the more accurate genes^27^. Lastly, the multivariable cox analysis was performed to identify the coefficient and p-value of ribosome-related gene. The prognostic risk score was calculated for each patient as follow: risk score = ∑ expression level of gene x coefficient. The low-risk and high-risk were divided by the best cut-off value (**Supplementary Table S1**).

To verify the Ribosome-based prognostic model, the univariate and multivariate Cox analysis were performed in GSE10846 training set. The Kaplan-Meier (K-M) survival curves was used to test this model in different groups of GSE10846 and validated sets including GSE11318, GSE87371 and TCGA-DLBCL cohorts. The sensitivity and specificity of the model were examined by ROC curve analysis.

### Establishment of the Nomogram

The prognostic nomogram was constructed to predict the prognostic risk of DLBCL patients in 1, 3 and 5 years by the rms R package. The independent parameters included age, gender, IPI score and risk score. The Calibration plots was applied to show the consistency between the predicted and factual OS in 1, 3 and 5 years.

### Predicting Drug Response

The responses of DLBCL patients to a range of chemotherapeutic and targeted drugs, were analyzed by the pRRophetic R package. Then the boxplots and Wilcoxon rank test were used to exhibit the difference between low and high-risk score groups in GSE10846 cohort (**Supplementary Table S1**).

### GSEA

Gene set enrichment analysis (GSEA) was performed to find the related pathways between low and high-risk score groups in GSE10846 cohort. Pathways with a p-value <0.05 and false discovery rate (FDR) <0.25 were thought significantly enriched (**Supplementary Table S1**).

### Knocking out NLE1

The cell lines of DLBCL including OCI-LY7, TMD8 and 293T were purchased from the American Type Culture Collection (ATCC). We packaged lenti-virus by 293T cell. The sgRNA targeting NLE1 or negative control as follow: NLE1-sg1: TGAGCCGATACAACCTCGTG; NLE1-sg3: ACTGACTATGCCCTGCGCAC; Nontarget-sg: ACGGAGGCTAAGCGTCGCAA. After knocking out the NLE1, we tested the proliferation by the EdU staining.

### Statistical Analysis

All statistical analyses were performed by R statistical software version 4.0.4 and GraphPad Prism 8.0. The different Ribosome-related genes were screened by Wilcoxon test and Fisher's exact test. The boxplot in figure 3D were analyzed by unpaired test. The boxplots of in figure 4G-I, figure S1A-C, figure S3D-F and figure 11A-L were analyzed by the Wilcoxon test. Stratification variables in **Table 3** were compared by chi-square and Fisher's exact test. The K-M curves used the log-rank test to compare low and high risks groups. The bar plots of the percentage of proliferation in figure 13C were compared by unpaired t-test.

## Results

**Figure 1:**
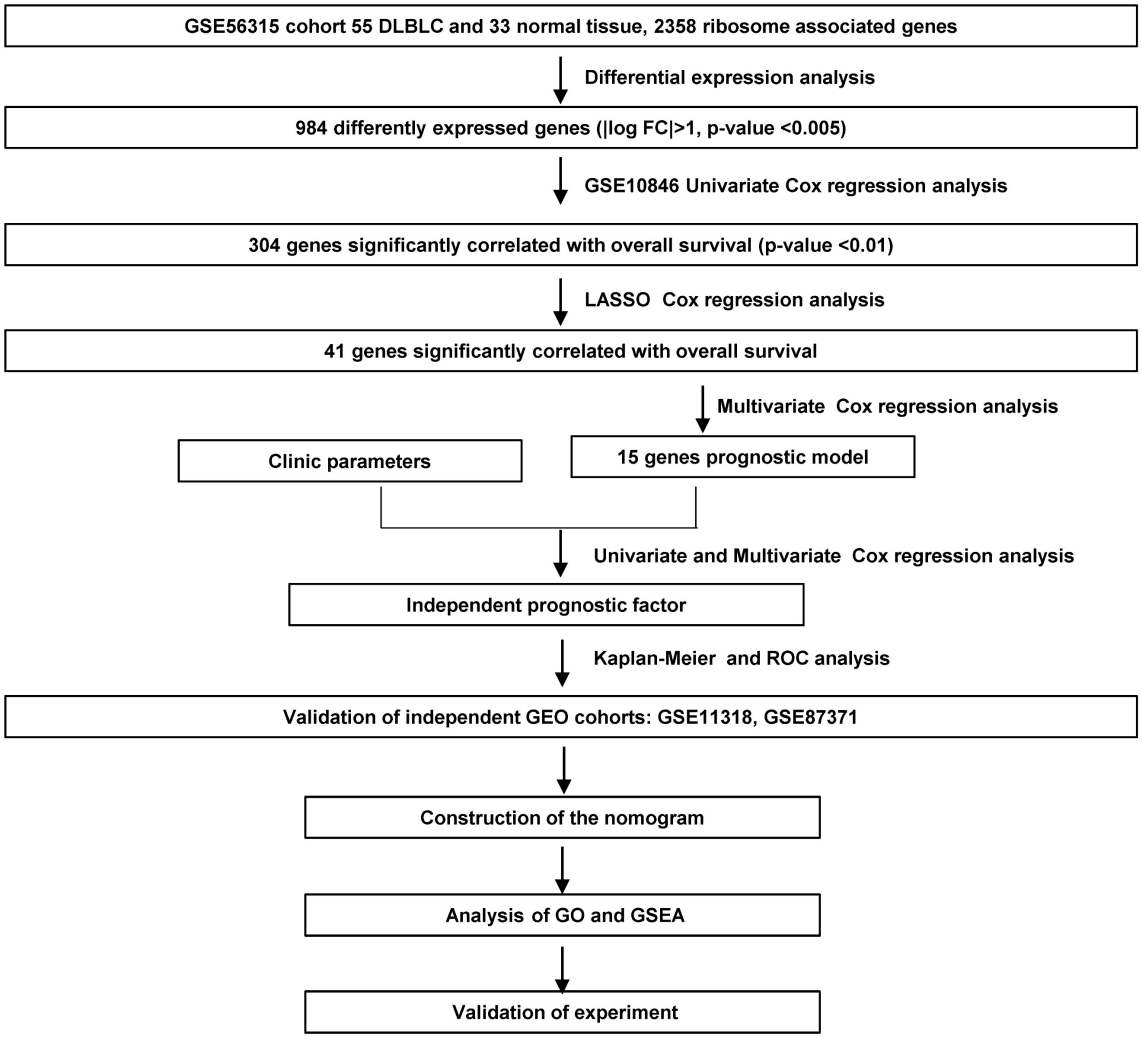
Flowchart of the study.

We screened the RibGs between 33 health donors and 55 DLBCL patients. Our training datasets included 414 DLBCL patients and the validation sets included 421 DLBCL patients. The detail information of these donors and patients were summarized in **Table 1**. The analyzed procedures were exhibited in **Figure 1**.

### Differentially Expressed Ribosome-related genes (RibGs) and Pathways

**Figure 2:**
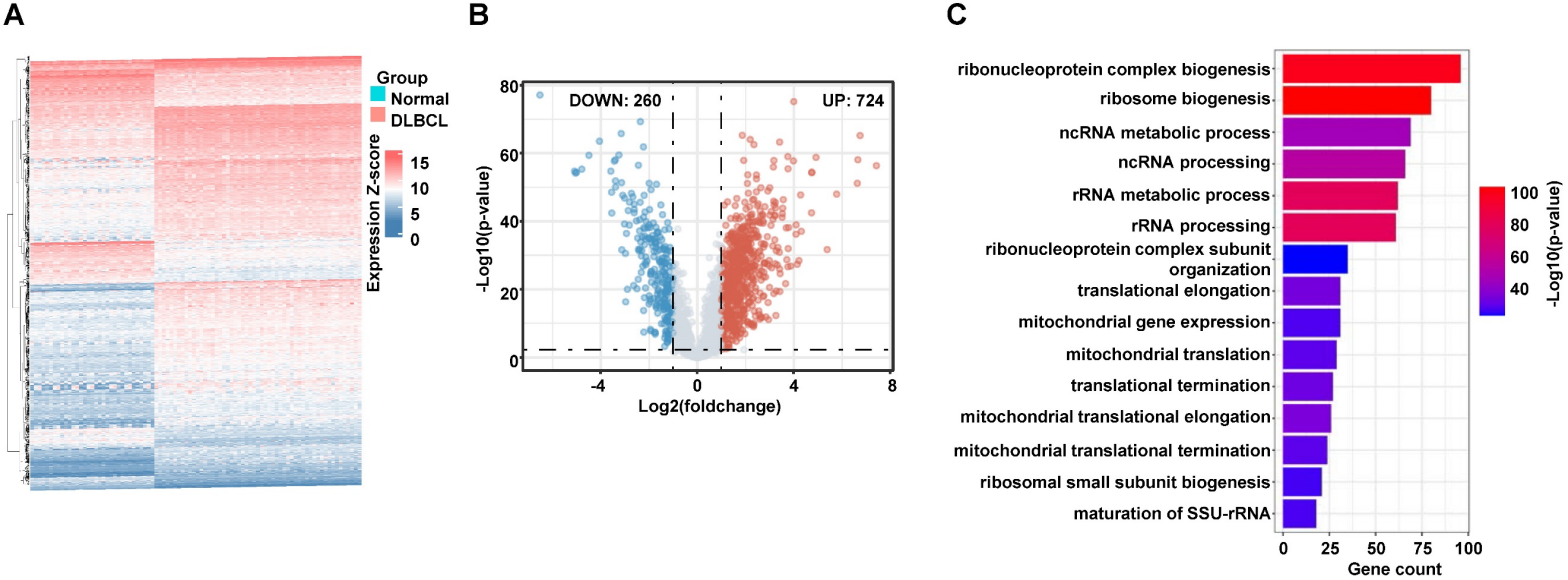
Screening of the differentially expressed RibGs. (A and B) The heatmap (A) and the Volcano plots (B) of the differentially expressed ribosome-related genes. (C) Bar plots of the enriched pathways by GO. RibGs: Ribosome related genes, GO: Gene Ontology.

To collect the RibGs, we downloaded 2592 RibGs from the GSEA database, and we selected the 2358 common genes among the GSEA and GSE datasets. Then, we compared the differentially expressed RibGs between the normal B cells and DLBCL B cells by limma package in GSE56315 set. There were 984 differentially expressed genes under the condition of Log2FC < -1 or > 1 and p < 0.005 (**Figure 2A**). The number of upregulated was 724 and downregulated was 260 in the DLBCL patients (**Figure 2B**). We analyzed the pathways enriched by the upregulated genes, and the bar plots showed the top 15 pathways associated with ribosomal pathways such as ribosome biogenesis, translational elongation and mitochondrial translation (**Figure 2C**). These data suggested that the RibGs could affect the cellular functions by mRNA translation and mitochondrial activities.

### Construction of the Prognostic RibGs signature

**Figure 3:**
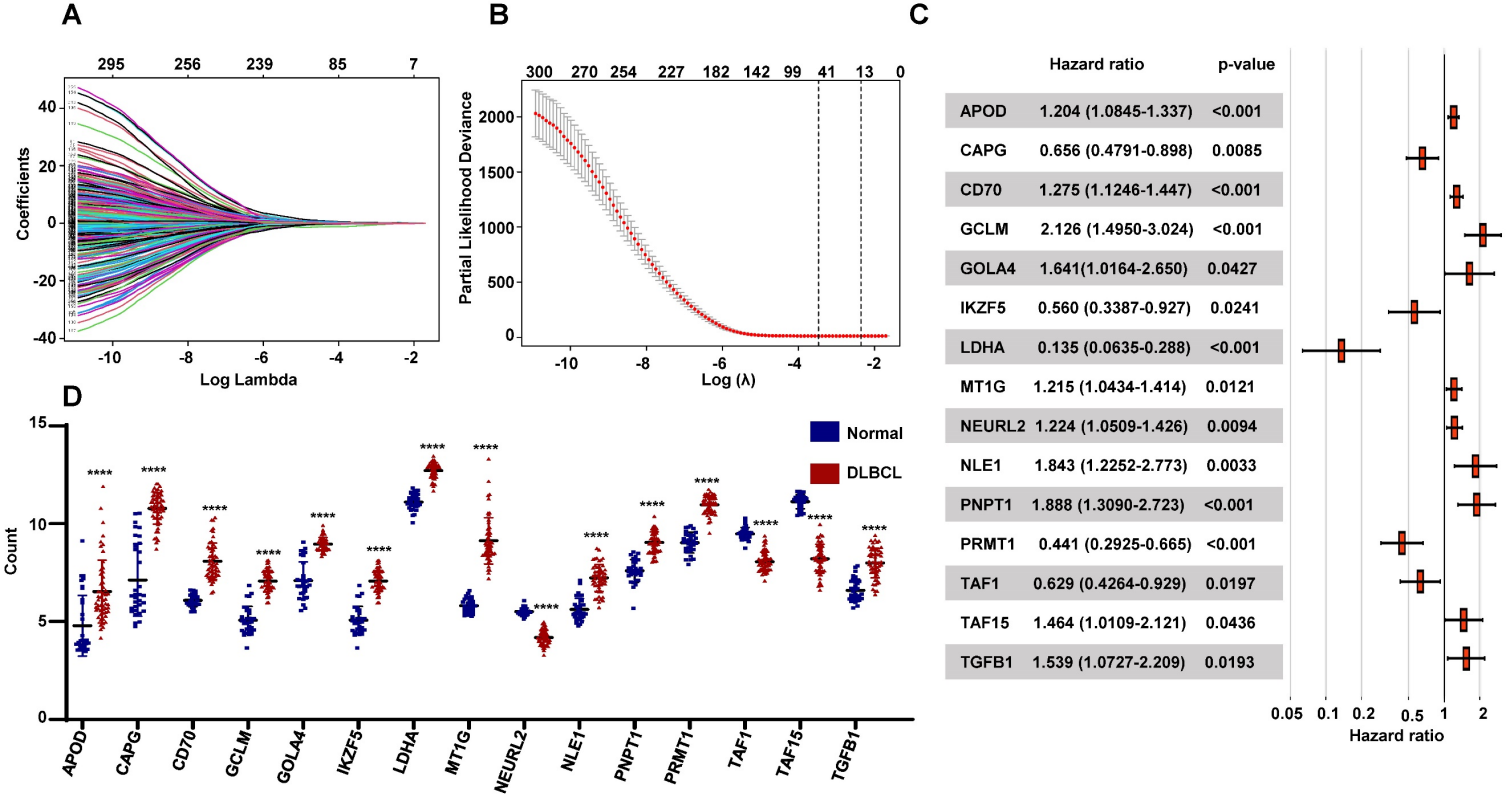
Establishment of the prognostic RibGs signature. (A) LASSO coefficient profile plots of the 304 prognosis-related genes. (B) Penalty plot for the LASSO model for the 304 prognostic genes with error bar denoting the standard errors. (C) Forest plots of the multivariate Cox regression analyses of the 15 gene significantly associated OS. (D) Expression level of the prognostic 15 genes between normal B cells and DLBCL B cells. P < 0.05: *, P < 0.01: **, P < 0.001: ***, P < 0.0001 ****.

To seek the prognostic genes, we performed univariate Cox regression analysis using the 984 genes and identified 304 genes significantly associated with OS. These genes were conducted LASSO Cox regression analysis, which selected 41 genes related with OS (**Figure 3A-B**). Then we applied multivariable cox analysis to find the genes of prognostic signature, which included 15 genes significantly associated with OS (**Figure 3C**). The prognostic risk score for each patient was calculated, basing on the expression levels of the 15 genes and the coefficients from multivariate Cox regression analyses, as follow: APOD x 0.186 + CAPG x (-0.422) + CD70 x 0.243 + GCLM x 0.754 + GOLGA4 x 0.495 + IKZF5 x (-0.579) + LDHA x (-2.0) + MT1G x 0.195 + NEURL2 x 0.202 + NLE1 x 0.611 + PNPT1 x 0.636 + PRMT1 x (-0.819) + TAF1 x (-0.416) +TAF15 x 0.381 +TGFB1 x 0.431. Moreover, the expressions of these genes were differentially expressed between normal donors and DLBCL patients significantly (**Figure 3D**), and their functions were vital to cellular activities such as reaction oxygen species, lymphoid development, ribosomal assembly and RNA polymerase II (**Table 2**).

**Figure 4:**
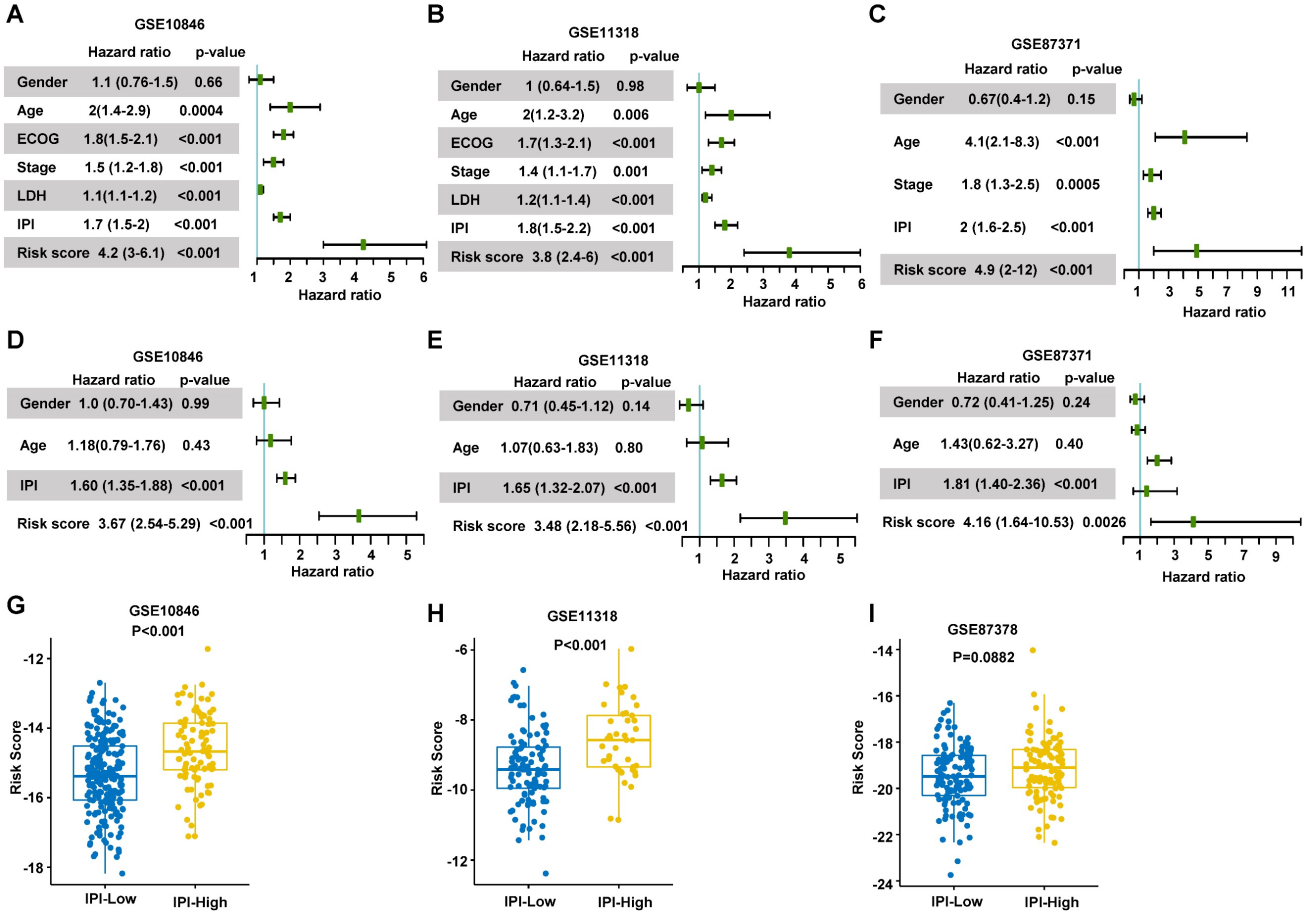
Validation of the prognosis model. (A-C) Forest plots of the univariate Cox regression analyses of clinical parameters and risk score in training dataset and validation dataset. (D-F) Forest plots of the multivariate Cox regression analyses of clinical parameters and risk score in training dataset and validation datasets. (G-I) Box plots of risk score in IPI-Low and IPI-High groups in training dataset and validation dataset. Training dataset: GSE10846; Validation datasets: GSE11318 and GSE87371; IPI-Low: 0-2, IPI-High: 3-5.

To validate the risk score, we conducted the univariate and multivariate Cox regression analyses to assess the predictive function of the RibGs model in training dataset and validation datasets. The analyses including gender, IPI score, age and risk score of the RibGs model, showed the risk score and IPI score correlated with OS significantly in univariate (**Figure 4A-C**) and multivariate Cox regression (**Figure 4D-F**). The IPI score is the most often used to stratify DLBCL patients into low risk (0-1), low intermediate risk (2), high intermediate risk (3) and high risk (4-5). The risk score of prognostic RibGs signature was higher in subgroup of high IPI score (3-5) than low IPI score (0-2) subgroup in the training set GSE10846 and validation sets (**Figure 4G-I**). Patients in GCB subgroup had a better prognosis than non-GCB subgroup, consistently, we found that the GCB patients had a lower risk score than non-GCB (**Figure S1A-C**). In summary, these data indicated that the risk score of the RibGs signature had a negative correlation with the favorable prognosis.

### Evaluation of the Prognostic RibGs Signature

**Figure 5:**
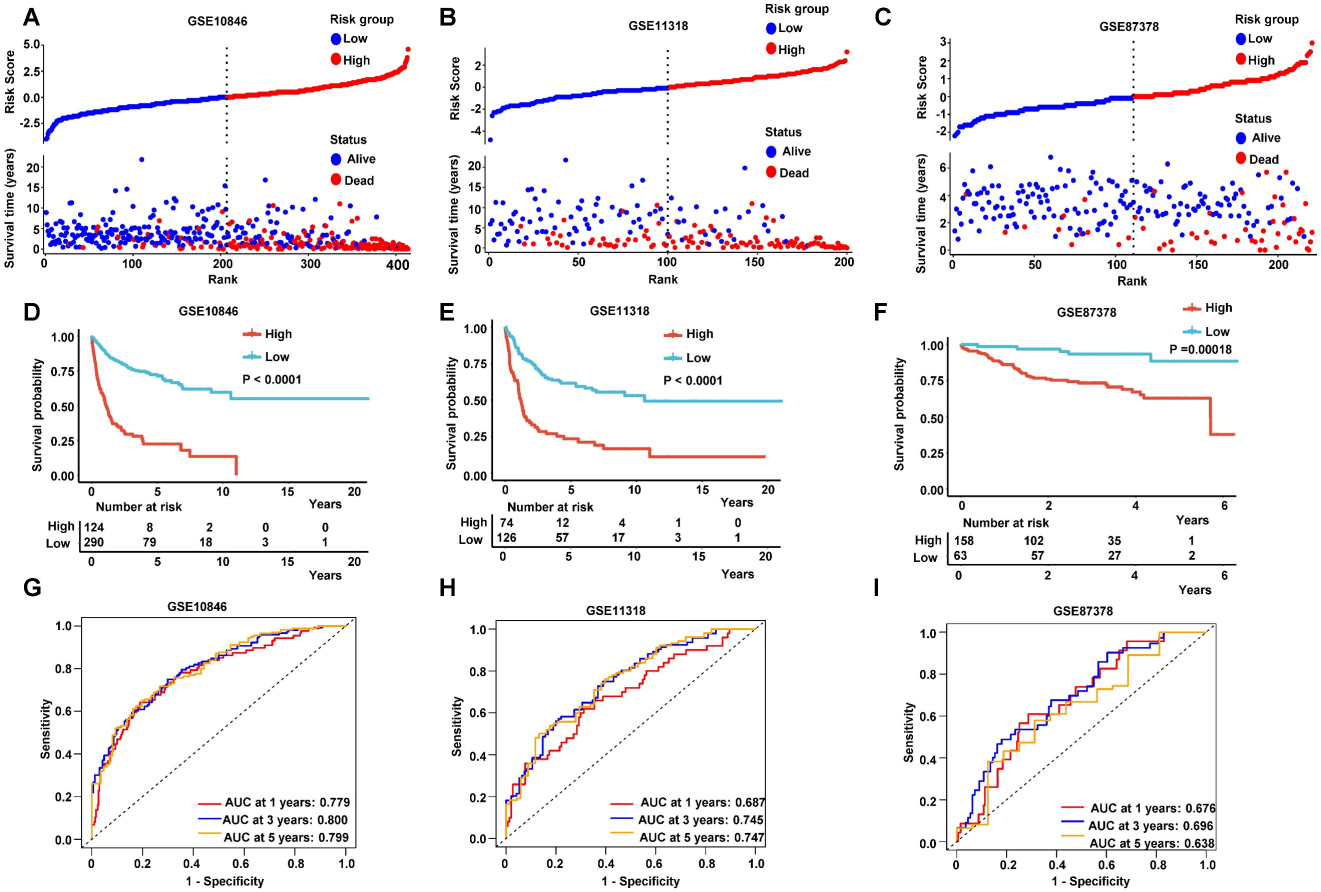
Prediction of the prognostic gene signature. (A-C) The distributions of the risk score, survival time, and status of patients in training cohorts and validation cohorts. (D-F) Kaplan-Meier curves of patients in the high-risk score group and low-risk score group for OS in the training set and validation sets. (G-I) The time-dependent ROC curves of the prognostic gene signature in training dataset and validation datasets. Training dataset: GSE10846; Validation datasets: GSE11318 and GSE87371.

The patients were divided into low-risk group and high-risk group by the median values of their risk scores. The correlation between the risk scores and survival statuses were exhibited by scatterplots in training set and validation sets (**Figure 5A-C**). The low-risk patients defined by the best cutoff, had a significantly higher survival probability whatever in training dataset or in validation datasets (**Figure 5D-F and Figure S2A**). The specificity and sensitivity of the signature were pretty good, which were reflected by the area under the receiver operating characteristic (ROC) curve (AUC) for predicting 1, 3, and 5 years in training dataset and validation datasets (**Figure 5H-I and Figure S2B**). These data suggested that the RibGs signature was reliable to predict the prognosis of DLBCL patients.

### The Predictive capability of RibGs Signature in different Subgroups of DLBCL

**Figure 6:**
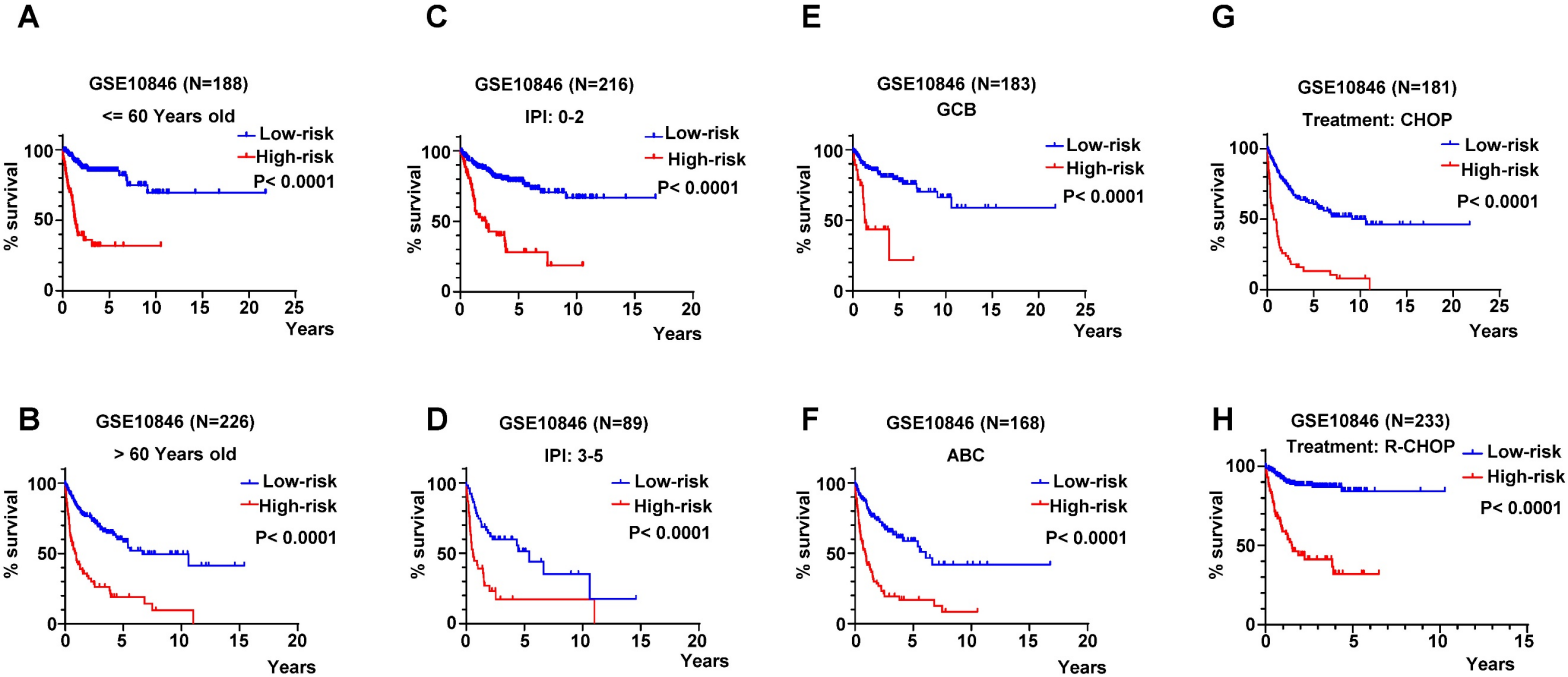
The K-M curves in different subgroups. (A-B) The K-M curves in ages of patients younger and older than 60-year-old. (C-D) The K-M curves of prognosis model in low-IPI score group and high-IPI score group in training dataset GSE10846. (E-F) The K-M curves of low-risk score and high-risk score in GCB and non-GCB patients in GSE10846 dataset. (G-H) The K-M curves of low-risk score and high-risk score in CHOP and R-CHOP treated patients.

The age is a key influence on prognosis, for instance, patients under 60-year-old always had more favorable prognoses than older patients. Therefore, we validated the RibGs prognostic model in patients under 60-year-old and older than 60-year-old patients. The patients of high-risk of the RibGs model showed more shorter OS time than low-risk patient in both age subgroups (**Figure 6A-B**). IPI score is extremely important to classify DLBCL patients into different risk groups, thus, we examined the prognostic model in low score of IPI (0-2) and high score of IPI (3-5) subgroups dividedly. The high-risk patients had a shorter survival time in low IPI score subgroup as well as in high IPI score subgroup (**Figure 6C-D**). Similarly, not only could the prognostic model predict the prognoses in GCB and non-GCB subgroups significantly (**Figure 6E-F**), but also forecast the prognoses of different treatments of CHOP and R-CHOP subgroups (**Figure 6G-H**) in the training set. Above data illustrated that our prognostic model could distinguish the heterogeneity of the DLBCL patients in different subgroups.

### Comparing the RibGs model with clinical parameters

**Figure 7:**
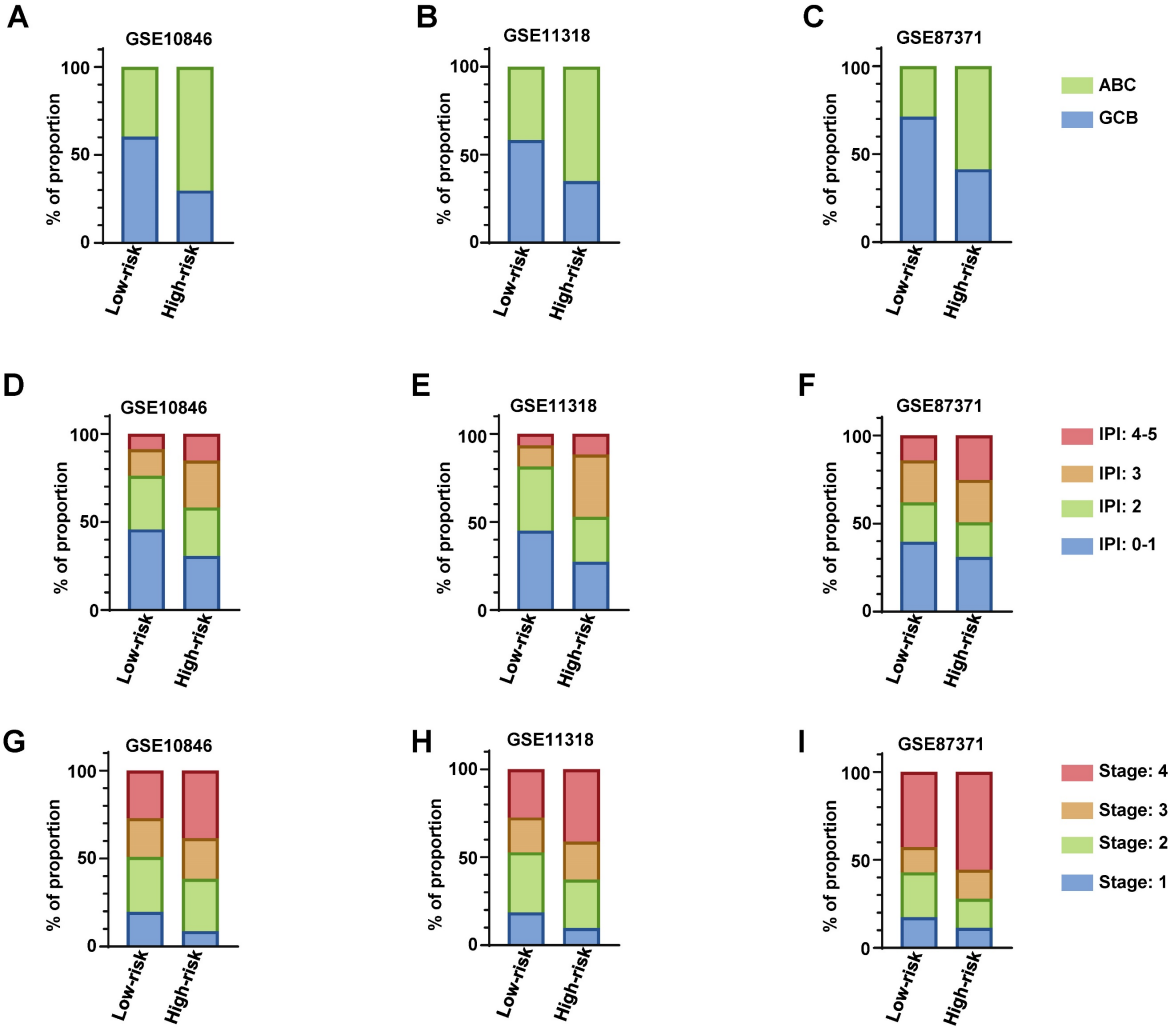
The distribution of clinical parameters between low-risk and high-risk subgroups. (A-C) The stacked bar plots of the GCB and ABC classified patients in low-risk and high-risk subgroups in training dataset and validation datasets. (D-E) The stacked bar plots of IPI score in low- and high-risk subgroups in training dataset and validation datasets. (G-I) The proportion of the stage of DLBCL in low- and high-risk subgroups in training dataset and validation datasets. Training dataset: GSE10846, validation datasets: GSE11318 and GSE87371.

We descripted the relationship of risk score of the RibGs model with the clinical factors including age, gender, GCB/ABC, stage, LDH, ECOG, IPI and status in **Table 3**. The risk score was significantly correlated with GCB/ABC, stage, ECOG, IPI and stage in training dataset and validation datasets. Furthermore, as we all known, the DLBCL patient of ABC classification had a worse prognosis than GCB. we found that high-risk subgroup had more proportion of ABC patients than low-risk subgroup in training dataset and validation datasets (**Figure 7A-C**). Consistently, the higher score of IPI (3 and 4-5) subgroup had a larger proportion of patients with high-risk than low-risk in training dataset and validation datasets (**Figure 7D-F**). In line with the above results, the stage of the DLBCL patients, showed more advanced in high-risk subgroup than low-risk subgroup (**Figure 7G-I**). Next, we compared the consistency between the risk score and the R-IPI, which showed that the high-risk group patients had a higher proportion of R-IPI with score of 3-5 (Figure S3A-S3C). Similarly, the higher score of R-IPI, the higher risk score was (**Figure S3D-S3F**) in the training dataset and validation datasets. Lastly, we analyzed the correlation between the risk score and different age or stage associated with the NCCN-IPI. Consistently, the high-risk patients had larger percentage of older years (**Figure S4A-S4C**) or advanced patients (**Figure S4D-S4F**). In conclusion, these results exhibited that our RibGs model had a marked consistency with the present clinical evaluate factors.

### RibGs model acted as a supplement to the IPI score

**Figure 8:**
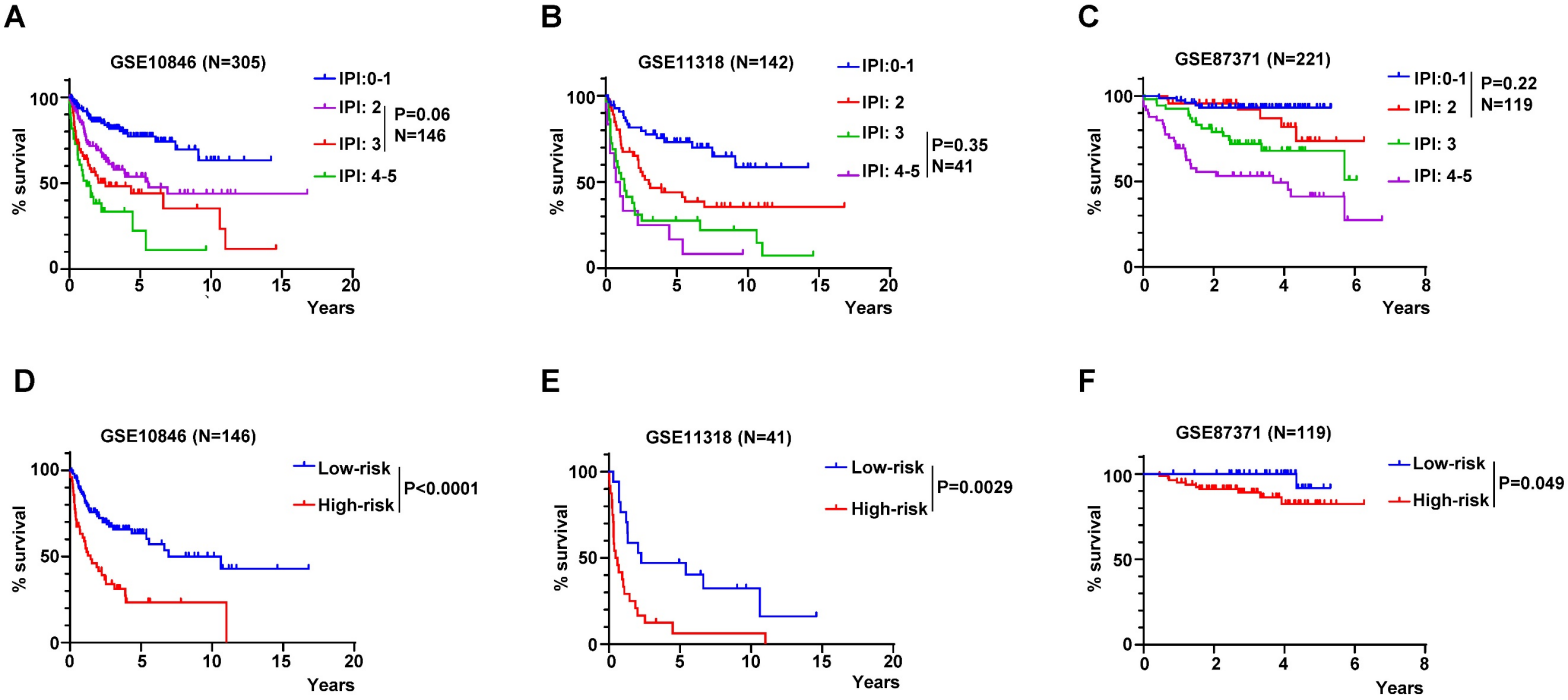
The RibGs model discriminated the prognosis of DLBCL patients accurately. (A-C) The K-M curves of patients of different IPI scores in training dataset and validation datasets. (D-F) The K-M curves of the no significance patients in the IPI score were based on the risk score of the RibGs model in training dataset and validation datasets. Training dataset: GSE10846, validation datasets: GSE11318 and GSE87371.

The IPI score is used mostly in clinical to assess the prognosis of DLBCL patient. However, the IPI score couldn't discriminate each patient accurately, for example, the K-M curves of the IPI score of 2 and 3 were overlapped in GSE10846, but the risk score of the RibGs model distinguished the survivals of these patients significantly (**Figure 8A and 8D**). Similarly, the IPI score of 3 and 4-5 couldn't predict the survival accurately, in contrast, these patients assessed by the risk score of the RibGs model had an outstanding predictive ability in GSE11318 (**Figure 8B and 8E**). In addition, the K-M curves of GSE87371 of different IPI scores and risk score had the similar results (**Figure 8C and 8F**). Above data showed that the RibGs model predicted the prognoses accurately of patients with less distinction by IPI, therefore, this model could be acted as a supplement to the IPI in the future.

### Establishment of the Nomogram

**Figure 9:**
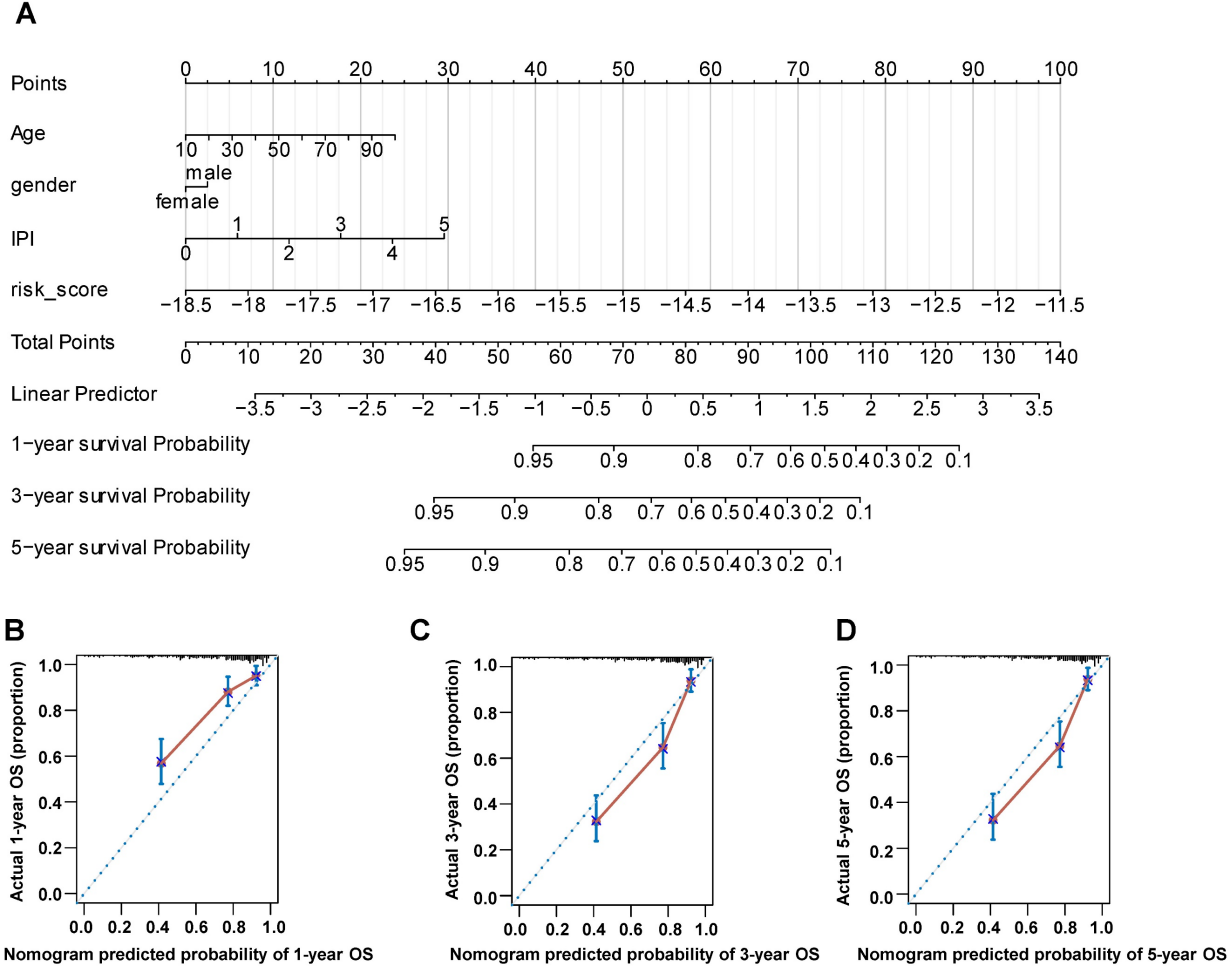
The nomogram was constructed using gender, age, IPI score and risk score to predict the OS. (A) The prognostic nomogram predicted the OS in 1, 3 and 5 years in training dataset. (B-D) The calibration curves for internal validation of the nomogram predicting 1-, 3-, and 5-year OS.

The nomogram is a useful tool, which combines some key clinical factors to predict the prognosis accurately. Therefore, we constructed the nomogram using the age, gender, IPI score and risk score to predict the survival probability of 1, 3, and 5 years (**Figure 9A**) in training dataset. The risk score of our prognostic model contributed to the total points more largely. Then we validated the nomogram by the calibration curves, which suggested a reliable prediction of OS compared with actual OS at 1, 3 and 5 years (**Figure 9B-D**). Furthermore, we established the nomogram by age, gender and IPI and validated its' predictivity by the calibration curves (**Figure S5A-S5C**). Comparing these calibration curves from figure 9B-D and figure S5A-S5C, we found that the nomogram containing the risk score performed better than the nomogram only including IPI to predict the prognoses of DLBCL patients.

### Enrichment of pathways

**Figure 10:**
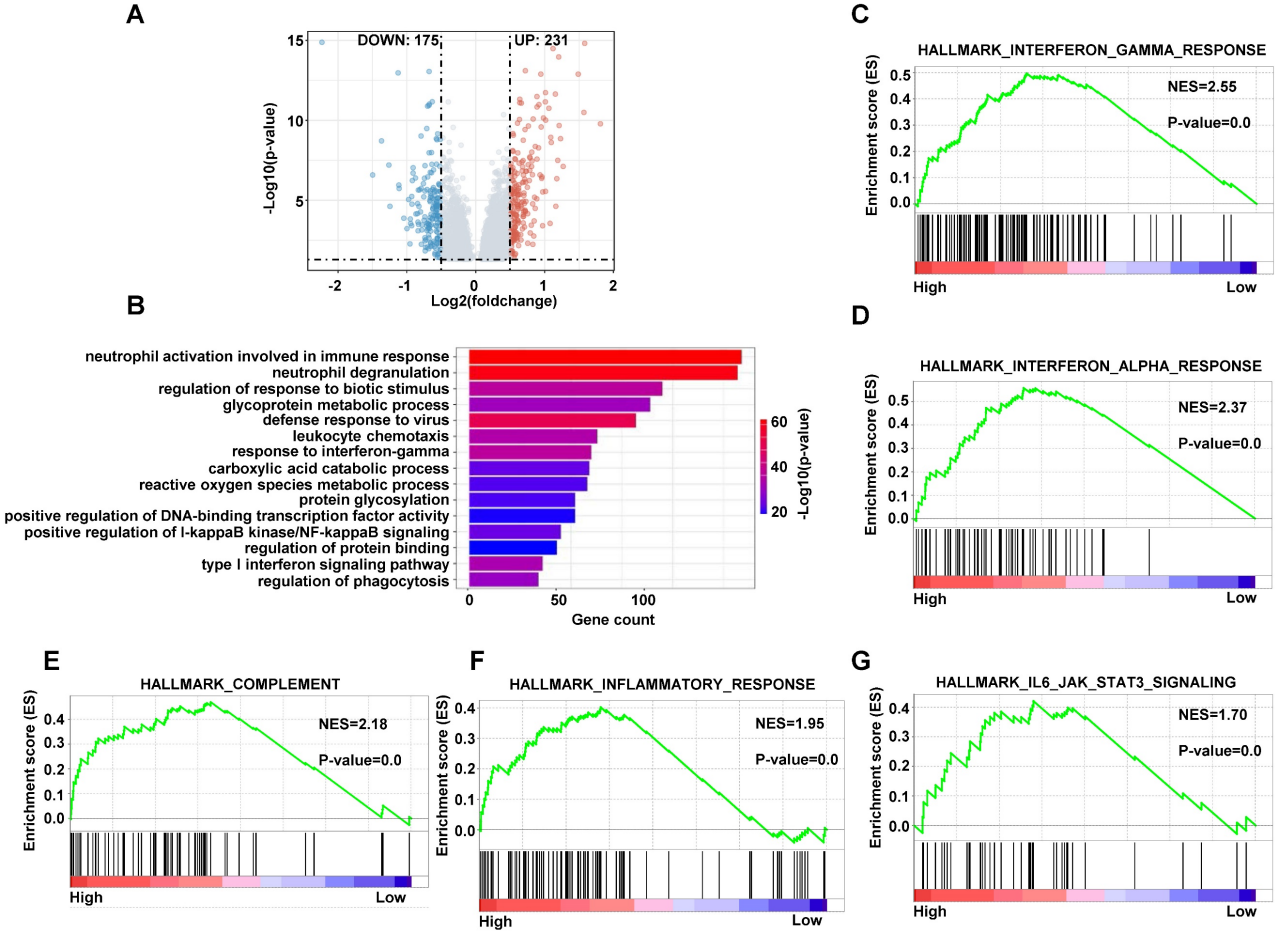
The enriched pathways in high-risk score group. (A) The volcano plots of differentially expressed genes in high-risk group compared with low-risk group. (B) The bar plots of GO upregulated pathways in high-risk score in training dataset. (C-G) The GSEA of upregulated pathways in high-risk score group in training dataset.

In order to analyze the different cellular pathways between high-risk patients and low-risk patients, we performed gene differentially expressed analysis by limma package in training dataset. There were 231 genes upregulated and 175 genes downregulated under the condition of p < 0.05, Log2FC > 0.5 or < -0.5 in high-risk group compared with low-risk group (**Figure 10A**). We conducted the gene ontology (GO) analysis using the upregulated genes in high-risk patients by the clusterprofiler package. The pathways included immune response, glycoprotein metabolic process and reactive oxygen species metabolic process (**Figure 10B**). The GSEA analyze the pathways using the upregulated and downregulated genes simultaneously, so we applied the GSEA to analyze the different pathways using genes with p < 0.05. The interferon gamma response, complement, inflammatory response and IL6-JAK-STAT3 pathways were on the top of the analyses (**Figure 10C-10G and S6A-S6E**). These data indicated that the high-risk DLBCL patients had more active innate immune response.

### Responses to the common anti-tumor drugs between low-risk and high-risk patients

**Figure 11:**
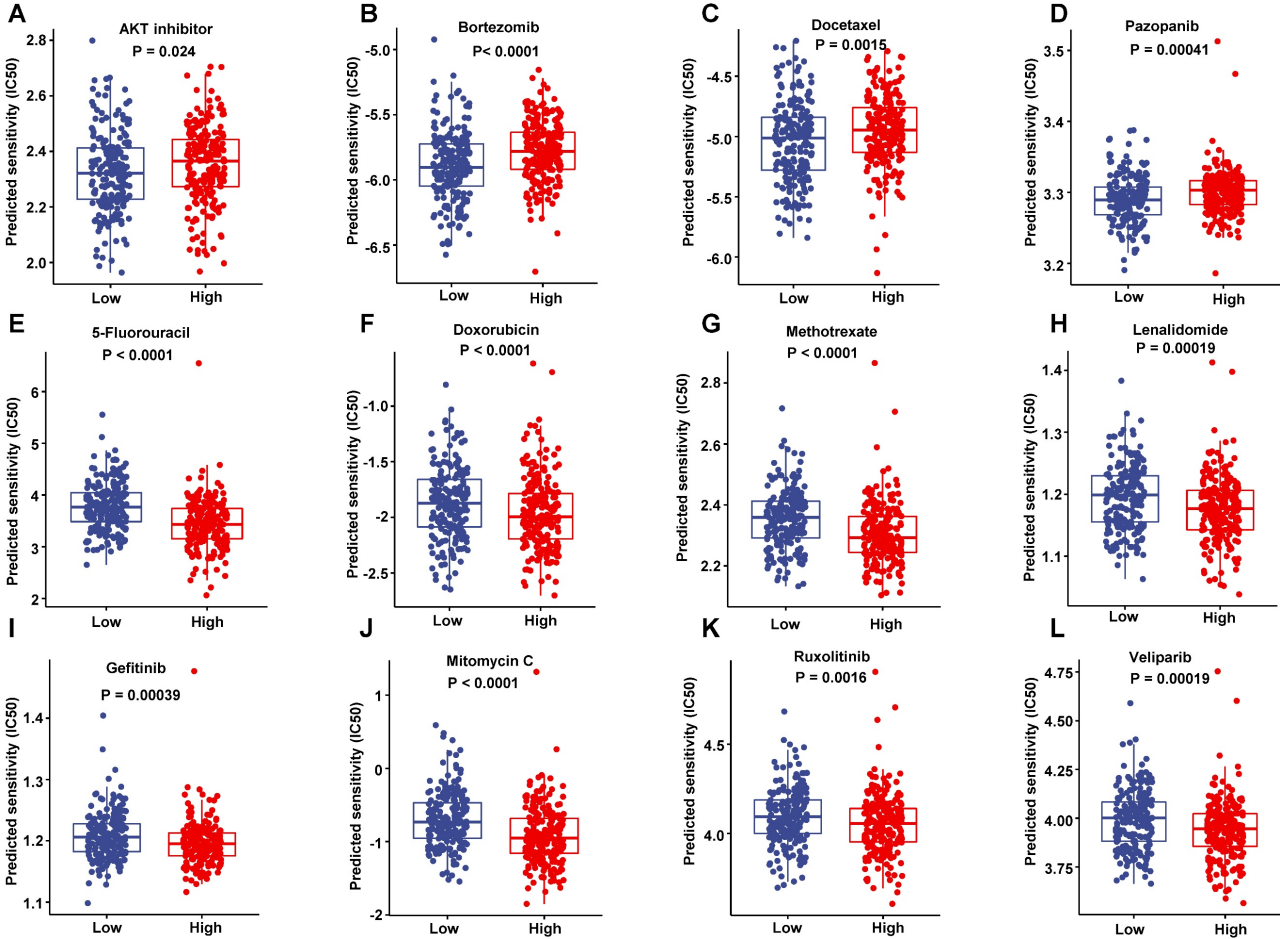
Sensitivity to drugs. (A-D) The drugs were more sensitive to low-risk score patients in the training dataset. (E-L) The drugs were more sensitive to high-risk score patients in the training dataset.

To improve the prognosis of the DLBCL patient, we predicted each patient's response to a range of drugs basing on their gene expression profiling by the pRRophetic package in training set. The low-risk patients were more sensitive to AKT inhibitor, Bortezomib, Docetaxel and Pazopanib than high-risk patients (**Figure 11A-D**). However, the high-risk patients had a lower half maximal inhibitory concentration (IC50) in a number of chemotherapeutics such as 5-Fluoroucacil, Doxorubicin, Methotrexate and lenalidomide (**Figure 11E-H**), as well as a lot of targeting inhibitors including Gefitinib, Mitomycin C, Ruxolitinib and veliparib (**Figure 11I-L**). These data suggested that these drugs may improve the treated outcomes of DLBCL patients potentially.

### Immunohistochemistry Staining of the prognostic proteins between normal and patients' nodes tissue

**Figure 12:**
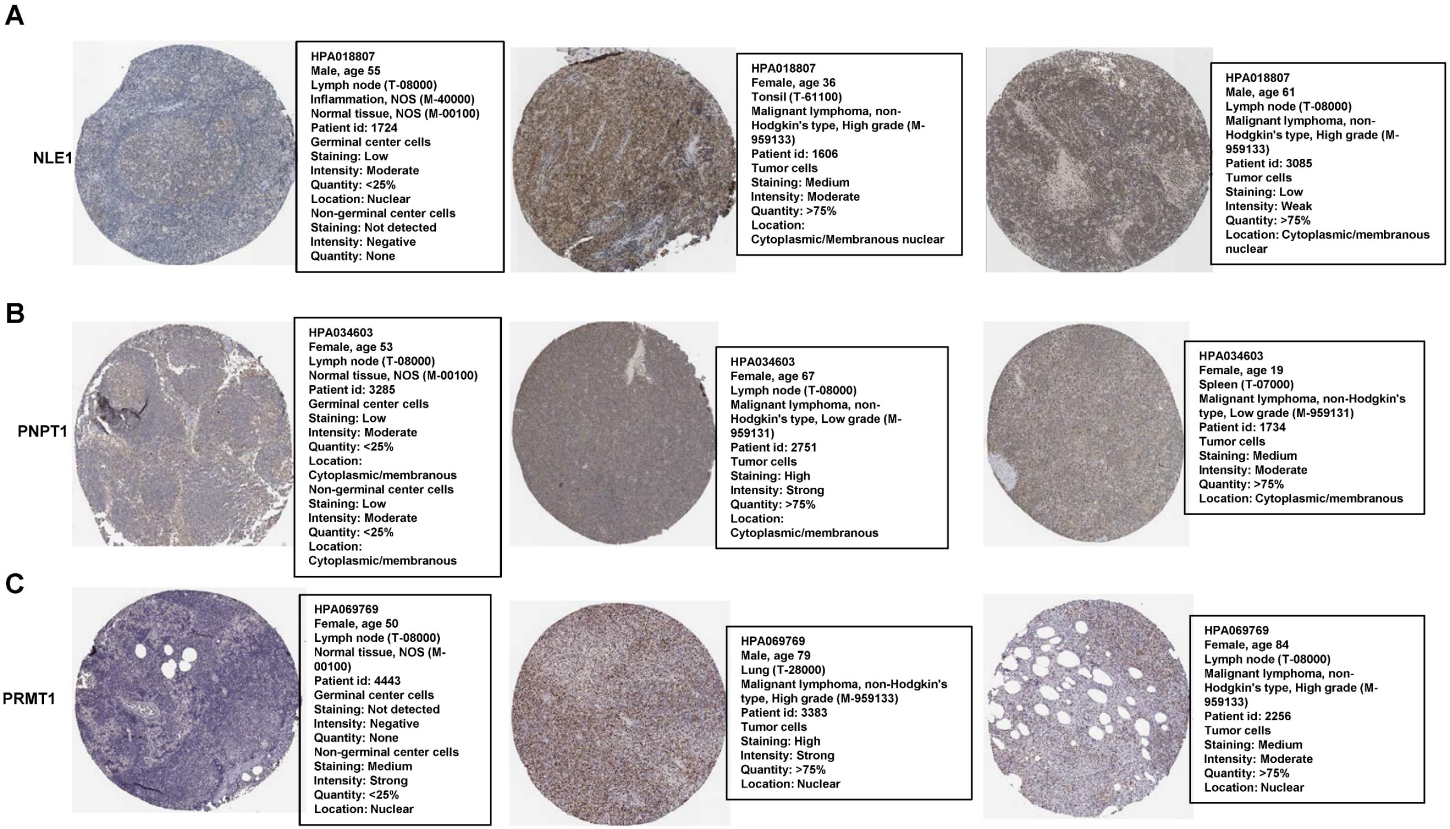
The proteins level of the prognostic genes. (A-C) The immunohistochemical of the NLE1, PNPT1 and PRMT1 from The Human Protein Atlas database.

Most of genes were based on their proteins to work in many biological processes finally. Therefore, we compared some proteins of the RibGs model including NLE1, PNPT1 and PRMT1 between normal and DLBCL patients' nodes, which exhibited higher level of these proteins in patients than healthy donors (**Figure 12A-C**), for each protein choosing the same antibody between health and patients from the HPA database. These data indicated that the prognostic genes and their proteins were highly expressed in DLBCL patients, which implied the unfavorable prognoses.

### CRISPR Screening the essential genes

**Figure 13:**
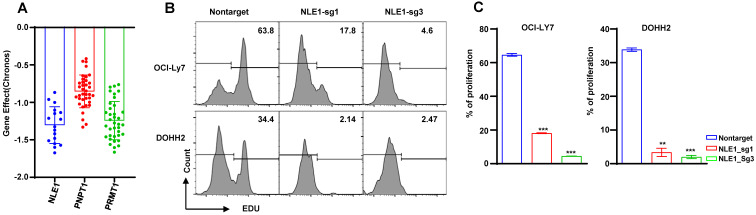
The dependency of the prognostic genes. (A) The bar plots of the NLE1, PNPT1 and PRMT1 genes' effect in DepMap database. (B-C) The analysis of proliferation by the EdU staining of the knocking out NLE1 and nontarget control.

In order to seek the promising target gene of DLBCL patient, we screened the dependency of these prognostic genes in DLBCL cells by DepMap database. We found the DLBCL cell lines were extremely dependent on these genes such as NLE1, PNPT1 and PRMT1 (**Figure 13A**), so we knocked out the NLE1 to validate the reliability by CRISPR mediated sgRNA in the DLBCL cell lines OCI-Ly7 and DOHH2. The proliferation was inhibited prominently in cells of knocking out the NLE1(**Figure 13B -C**). In conclusion, these experiments declared that we could target the prognostic gene NLE1 to therapy the DLBCL patient, especially in high-risk patient.

## Discussion

DLBCL is a heterogeneous disease including therapeutic response and OS time. At present, we have some criteria to stratify DLBCL patients of different risks, such as IPI score (29), GCB or ABC based on cell-of-origin (COO)^29^ and whether belonging to high-grade DLBCL^6^. However, these criteria ignore the abundant gene-expression information that is highly related to therapeutic responses and survival time. In this study, we constructed a prognostic model using the RibGs, which could discriminate the outcome of each DLBCL patient regardless of IPI score, GCB or ABC and treatment of CHOP or R-CHOP. It is the first time to combine prognosis of DLBCL with RibGs. We selected 15 RibGs to build the prognostic signature through Cox regression and lasso analyses. The functions of the15 RibGs include cell development, ribosome assembly and transcription, which are all associated with survival probability significantly. Moreover, the signature stratified survival time in training dataset as well as in validation datasets powerfully.

Recently, many reports revealed the correlation between the gene expressed profiling and survival time in DLBCL^30^, ^31^ or other tumors^32^, ^33^, but they only used one or two biological functions. However, ribosome participates in numerous bioprocesses such as translation, proliferation and tumorigenesis, we chose the RibGs to develop the prognostic model including these many cellular functions. Moreover, we found that the RibGs model could distinguish survival of some patients, whom the IPI score discriminated difficultly. Then the GSEA enriched pathways showed that the innate immune responses such as interferon, complement and inflammatory responses, were upregulated in high-risk group. Besides, we screened some drugs including methotrexate, lenalidomide and parp1 inhibitor that were more sensitive to high-risk DLBCL patients. Importantly, knocking out the upregulated gene NLE1 inhibited cell proliferation markedly, which provided a gene to target in the future therapy of DLBCL patient.

Nevertheless, we should take several limitations of our prognostic model into consideration. On the one hand, the clinical information of these cohorts was limited and incomplete, for example the reaction to therapy didn't illustrate. Therefore, we couldn't compare the consistency between the risk and responses to clinical treatments. On the other hand, the risk of the prognostic model needs to further validate. Importantly, the target of the NLE1, should study comprehensively.

Come into a conclusion, we constructed a reliable prognostic RibGs signature that associated the risk of each DLBCL patient with survival time significantly, which could be an ideal complement to the IPI score. Furthermore, our finding provided new therapeutic strategy such as targeting NLE1 gene or functions associated with ribosome for relapsed or refractory DLBCL patient.

## Supplementary Material

Supplementary figures.

Supplementary table.

## Figures and Tables

**Table 1:** Clinical information of the datasets using in this study.

Cohort	GSE56315	GSE10846	GSE11318	GSE87371	TCGA
Number of patients Normal/ DLBCL	33/55	0/414	0/200	0/221	0/48
Age (y)	NA	62.5 (14-92)	64 (14-88)	60 (19-87)	NA
Gender Male/ Female/ NA	NA	172/ 224/ 18	110/ 90	116/ 105	NA
GCB/ ABC/ NA	NA	163/ 232/ 19	70/ 100/ 30	84/ 117/ 20	NA
Stage 1/ 2/ 3/ 4/ NA	NA	66/ 122/ 97/ 121/ 8	25/ 50/ 32/ 55/ 38	29/ 42/ 35/ 115	NA
LDH <1 / >1 / NA	NA	173/ 178/ 63	68/ 76/ 56	NA	NA
ECOG 0/ 1/ 2/ 3/ 4/ NA	NA	85/ 211/ 60/ 28/ 5/ 25	34/ 88/ 28/ 10/ 1/ 39	NA	NA
IPI 0/ 1/ 2/ 3/ 4/ 5/ NA	NA	45/ 81/ 90/ 56/ 28/ 5/ 109	15/ 40/ 46/ 29/ 12/ 58	31/ 43/ 45/ 53/ 35/ 14	NA
Status Alive/ Death	NA	249/165	88/112	168/53	39/9

**Table 2:** Functions of the 15 RibGs in the prognosis model

Gene	Function summary	Risk coefficient
APOD	response to reactive oxygen species	0.185732
CAPG	encodes a member of the gelsolin/villin family of actin-regulatory proteins	-0.421626
CD70	This cytokine is a ligand for TNFRSF27/CD27	0.243280
GCLM	limiting enzyme of glutathione synthesis	0.754312
GOLGA4	participates in glycosylation and transport of proteins and lipids in the secretory pathway	0.495399
IKZF5	implicated in the control of lymphoid development	-0.579333
LDHA	catalyzes the conversion of L-lactate and NAD to pyruvate and NADH in the final step of anaerobic glycolysis	-2.000347
MT1G	Enables zinc ion binding activity	0.194525
NEURL2	the adaptor component of the E3 ubiquitin ligase complex in striated muscle, and it regulates the ubiquitin-mediated degradation of beta-catenin during myogenesis	0.202417
NLE1	involved in Notch signaling pathway and ribosomal large subunit assembly	0.611415
PNPT1	implicated in RNA processing and degradation	0.635507
PRMT1	encodes a member of the protein arginine N-methyltransferase (PRMT) family	-0.818692
TAF1	Initiation of transcription by RNA polymerase II	-0.463185
TAF15	plays a role in RNA polymerase II gene transcription as a component of a distinct subset of multi-subunit transcription initiation factor TFIID complexes	0.381328
TGFB1	encodes a secreted ligand of the TGF-beta (transforming growth factor-beta) superfamily of proteins	0.431421

**Table 3:** The association among the risk score of the RibGs model and Clinical parameters.

Cohort	GSE10846			GSE11318			GSE87371		
Risk score	Low	High	P-value	Low	High	P-value	Low	High	P-value
Age (y) ≤60/ >60	104/ 111	35/ 56	0.13	41/ 50	18/ 33	0.29	31/ 32	78/ 80	0.99
Gender Male/ Female	128/ 86	43/ 48	0.045	55/ 36	21/ 30	0.035	31/ 32	85/ 73	0.55
GCB/ ABC/ NA	111/ 73/ 30	22/ 52/ 17	<0.0001	46/ 33/ 13	14/ 26/ 11	0.020	37/ 15/ 12	48/ 68/ 42	0.0004
Stage 1/ 2/ 3/ 4	42/ 67/ 47/ 58	8/ 27/ 21/ 35	0.01	17/ 31/ 18/ 25	5/ 14/ 11/ 21	0.044	11/ 16/ 9/ 27	18/ 26/ 26/ 88	0.04
LDH ≤1 / >1	115/ 99	42/ 49	0.26	46/ 45	21/ 30	0.3	NA	NA	NA
ECOG 0/ 1/ 2/ 3/ 4	55/ 117/ 26/ 13/ 3	13/ 45/ 22/ 9/ 2	0.003	25/ 50/ 14/ 2/ 0	6/ 27/ 11/ 6/ 1	0.0037	NA	NA	NA
IPI 0-1/ 2/ 3/ 4-5	98/ 65/ 32/ 19	28/ 25/ 24/ 14	0.013	41/ 33/ 11/ 6	14/ 13/ 18/ 6	0.0024	25/ 14/ 15/ 9	49/ 31/ 38/ 40	0.073
Status Alive/ Death	154/ 60	29/ 62	<0.0001	54/ 37	8/ 43	<0.0001	58/ 5	110/ 48	0.0002

## References

[1] Fisher RI, Miller TP, O'Connor OA (2004). Diffuse aggressive lymphoma. Hematology American Society of Hematology Education Program.

[2] Jemal A, Siegel R, Xu J, Ward E (2010). Cancer statistics, 2010. CA: a cancer journal for clinicians.

[3] Sung H, Ferlay J, Siegel RL, Laversanne M, Soerjomataram I, Jemal A (2021). Global Cancer Statistics 2020: GLOBOCAN Estimates of Incidence and Mortality Worldwide for 36 Cancers in 185 Countries. CA: a cancer journal for clinicians.

[4] Morrison VA (2021). Frontline therapy with R-CHOP for diffuse large B-cell lymphoma: Where have we come (or not come)? A Perspective. Journal of geriatric oncology.

[5] Coiffier B, Sarkozy C (2016). Diffuse large B-cell lymphoma: R-CHOP failure-what to do?. Hematology American Society of Hematology Education Program.

[6] Swerdlow SH, Campo E, Pileri SA, Harris NL, Stein H, Siebert R (2016). The 2016 revision of the World Health Organization classification of lymphoid neoplasms. Blood.

[7] Sehn LH, Berry B, Chhanabhai M, Fitzgerald C, Gill K, Hoskins P (2007). The revised International Prognostic Index (R-IPI) is a better predictor of outcome than the standard IPI for patients with diffuse large B-cell lymphoma treated with R-CHOP. Blood.

[8] Zhou Z, Sehn LH, Rademaker AW, Gordon LI, Lacasce AS, Crosby-Thompson A (2014). An enhanced International Prognostic Index (NCCN-IPI) for patients with diffuse large B-cell lymphoma treated in the rituximab era. Blood.

[9] Alizadeh AA, Eisen MB, Davis RE, Ma C, Lossos IS, Rosenwald A (2000). Distinct types of diffuse large B-cell lymphoma identified by gene expression profiling. Nature.

[10] Rosenwald A, Wright G, Chan WC, Connors JM, Campo E, Fisher RI (2002). The use of molecular profiling to predict survival after chemotherapy for diffuse large-B-cell lymphoma. The New England journal of medicine.

[11] Ott G, Ziepert M, Klapper W, Horn H, Szczepanowski M, Bernd HW (2010). Immunoblastic morphology but not the immunohistochemical GCB/nonGCB classifier predicts outcome in diffuse large B-cell lymphoma in the RICOVER-60 trial of the DSHNHL. Blood.

[12] Flowers CR, Sinha R, Vose JM (2010). Improving outcomes for patients with diffuse large B-cell lymphoma. CA: a cancer journal for clinicians.

[13] Molina TJ, Canioni D, Copie-Bergman C, Recher C, Brière J, Haioun C (2014). Young patients with non-germinal center B-cell-like diffuse large B-cell lymphoma benefit from intensified chemotherapy with ACVBP plus rituximab compared with CHOP plus rituximab: analysis of data from the Groupe d'Etudes des Lymphomes de l'Adulte/lymphoma study association phase III trial LNH 03-2B. Journal of clinical oncology: official journal of the American Society of Clinical Oncology.

[14] Noller HF (2012). Evolution of protein synthesis from an RNA world. Cold Spring Harbor perspectives in biology.

[15] Narla A, Ebert BL (2010). Ribosomopathies: human disorders of ribosome dysfunction. Blood.

[16] Vlachos A, Rosenberg PS, Atsidaftos E, Kang J, Onel K, Sharaf RN (2018). Increased risk of colon cancer and osteogenic sarcoma in Diamond-Blackfan anemia. Blood.

[17] Alter BP, Giri N, Savage SA, Rosenberg PS (2018). Cancer in the National Cancer Institute inherited bone marrow failure syndrome cohort after fifteen years of follow-up. Haematologica.

[18] Taskinen M, Ranki A, Pukkala E, Jeskanen L, Kaitila I, Mäkitie O (2008). Extended follow-up of the Finnish cartilage-hair hypoplasia cohort confirms high incidence of non-Hodgkin lymphoma and basal cell carcinoma. American journal of medical genetics Part A.

[19] Fancello L, Kampen KR, Hofman IJ, Verbeeck J, De Keersmaecker K (2017). The ribosomal protein gene RPL5 is a haploinsufficient tumor suppressor in multiple cancer types. Oncotarget.

[20] Tzoneva G, Perez-Garcia A, Carpenter Z, Khiabanian H, Tosello V, Allegretta M (2013). Activating mutations in the NT5C2 nucleotidase gene drive chemotherapy resistance in relapsed ALL. Nature medicine.

[21] Hofman IJF, van Duin M, De Bruyne E, Fancello L, Mulligan G, Geerdens E (2017). RPL5 on 1p22.1 is recurrently deleted in multiple myeloma and its expression is linked to bortezomib response. Leukemia.

[22] Lawrence MS, Stojanov P, Mermel CH, Robinson JT, Garraway LA, Golub TR (2014). Discovery and saturation analysis of cancer genes across 21 tumour types. Nature.

[23] De Keersmaecker K, Atak ZK, Li N, Vicente C, Patchett S, Girardi T (2013). Exome sequencing identifies mutation in CNOT3 and ribosomal genes RPL5 and RPL10 in T-cell acute lymphoblastic leukemia. Nature genetics.

[24] Liu Y, Easton J, Shao Y, Maciaszek J, Wang Z, Wilkinson MR (2017). The genomic landscape of pediatric and young adult T-lineage acute lymphoblastic leukemia. Nature genetics.

[25] Orsolic I, Jurada D, Pullen N, Oren M, Eliopoulos AG, Volarevic S (2016). The relationship between the nucleolus and cancer: Current evidence and emerging paradigms. Seminars in cancer biology.

[26] Truitt ML, Ruggero D (2017). New frontiers in translational control of the cancer genome. Nature reviews Cancer.

[27] Tibshirani R (1997). The lasso method for variable selection in the Cox model. Statistics in medicine.

[28] Pelletier J, Thomas G, Volarević S (2018). Ribosome biogenesis in cancer: new players and therapeutic avenues. Nature reviews Cancer.

[29] Lenz G (2013). Novel therapeutic targets in diffuse large B-cell lymphoma. EJC supplements: EJC: official journal of EORTC, European Organization for Research and Treatment of Cancer [et al].

[30] Jiang Y, Sun H, Xu H, Hu X, Wu W, Lv Y (2022). Immunophenotypic Landscape and Prognosis-Related mRNA Signature in Diffuse Large B Cell Lymphoma. Frontiers in genetics.

[31] Hu J, Xu J, Yu M, Gao Y, Liu R, Zhou H (2020). An integrated prognosis model of pharmacogenomic gene signature and clinical information for diffuse large B-cell lymphoma patients following CHOP-like chemotherapy. Journal of translational medicine.

[32] He L, Chen J, Xu F, Li J, Li J (2020). Prognostic Implication of a Metabolism-Associated Gene Signature in Lung Adenocarcinoma. Molecular therapy oncolytics.

[33] Jiang Y, Ouyang W, Zhang C, Yu Y, Yao H (2021). Prognosis and Immunotherapy Response with a Novel Golgi Apparatus Signature-Based Formula in Lung Adenocarcinoma. Frontiers in cell and developmental biology.

