# Supplementary material for: Prognostic Significance of Ribosome-related Genes Signature in Diffuse Large B Cell Lymphoma
Source: J Cancer. 2023 Jan 22;14(3):403–16. doi: 10.7150/jca.80926 (PMC9969582; doi:10.7150/jca.80926)
Supplement: Supplementary file 1 — Supplementary figures. [file jcav14p0403s1.pdf]

Supplementary

Figure S1

(A-C) Box plots of the risk score between the GCB group and non-GCB group in training dataset and validation datasets.

Figure S2

(A) The Kaplan-Meier curves of the TCGA cohort between low-risk and high-risk group. (B) The time-dependent ROC curves in the TCGA cohorts.

Figure S3

(A-C) The stacked bar plots of R-IPI score in low-risk and high-risk groups.
(D-E) The box plots of risk score in different R-IPI score subgroups.

Figure S4

(A-C) The stacked bar plots of different ages subgroups according NCCN-IPI in low-risk and high-risk groups.
(D-F) The stacked bar plots of different stages subgroups classified by the NCCN-IPI in low-risk and high-risk groups.

Figure S5

(A-C) The calibration curves of the nomogram excluding the risk score at 1, 3 and 5 years.

Figure S6

(A-E) The GSEA plots analyzed by differentially expressed genes between high-risk and low-risk groups using the curated and ontology gene sets.

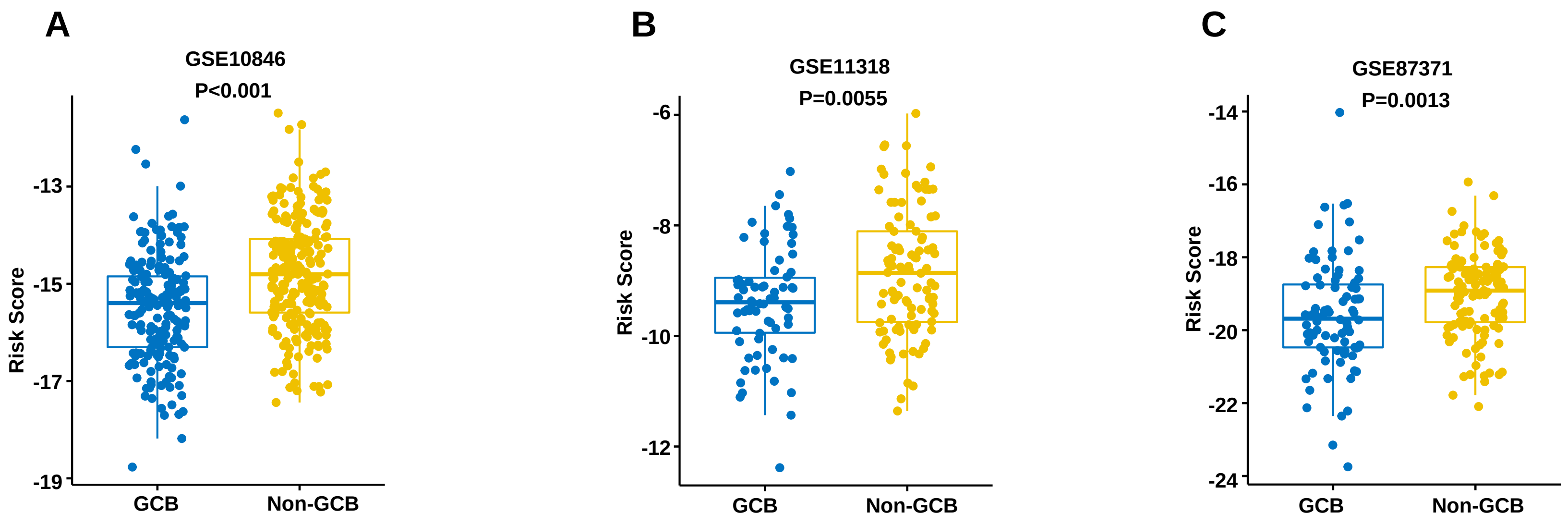
A
GSE10846
P<0.001
Risk Score
-13
-15
-17
-19
GCB
Non-GCB
B
GSE11318
P=0.0055
Risk Score
-6
-8
-10
-12
GCB
Non-GCB
C
GSE87371
P=0.0013
Risk Score
-14
-16
-18
-20
-22
-24
GCB
Non-GCB

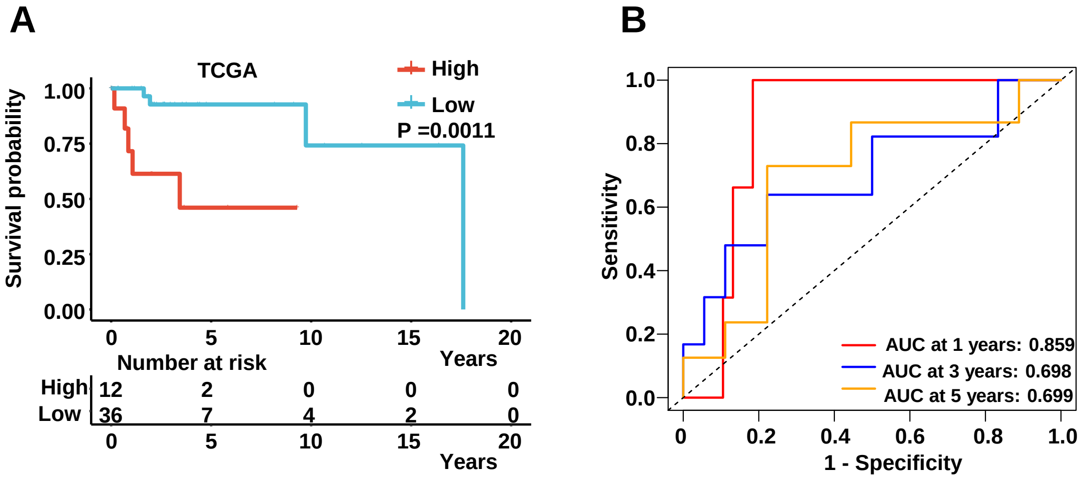
A
TCGA
High
Low
P =0.0011
Survival probability
1.00
0.75
0.50
0.25
0.00
0
5
10
15
20
Years
Number at risk
High 12 2 0 0 0
Low 36 7 4 2 0
0
5
10
15
20
Years
B
Sensitivity
1.0
0.8
0.6
0.4
0.2
0.0
AUC at 1 years: 0.859
AUC at 3 years: 0.698
AUC at 5 years: 0.699
0
0.2
0.4
0.6
0.8
1.0
1 - Specificity

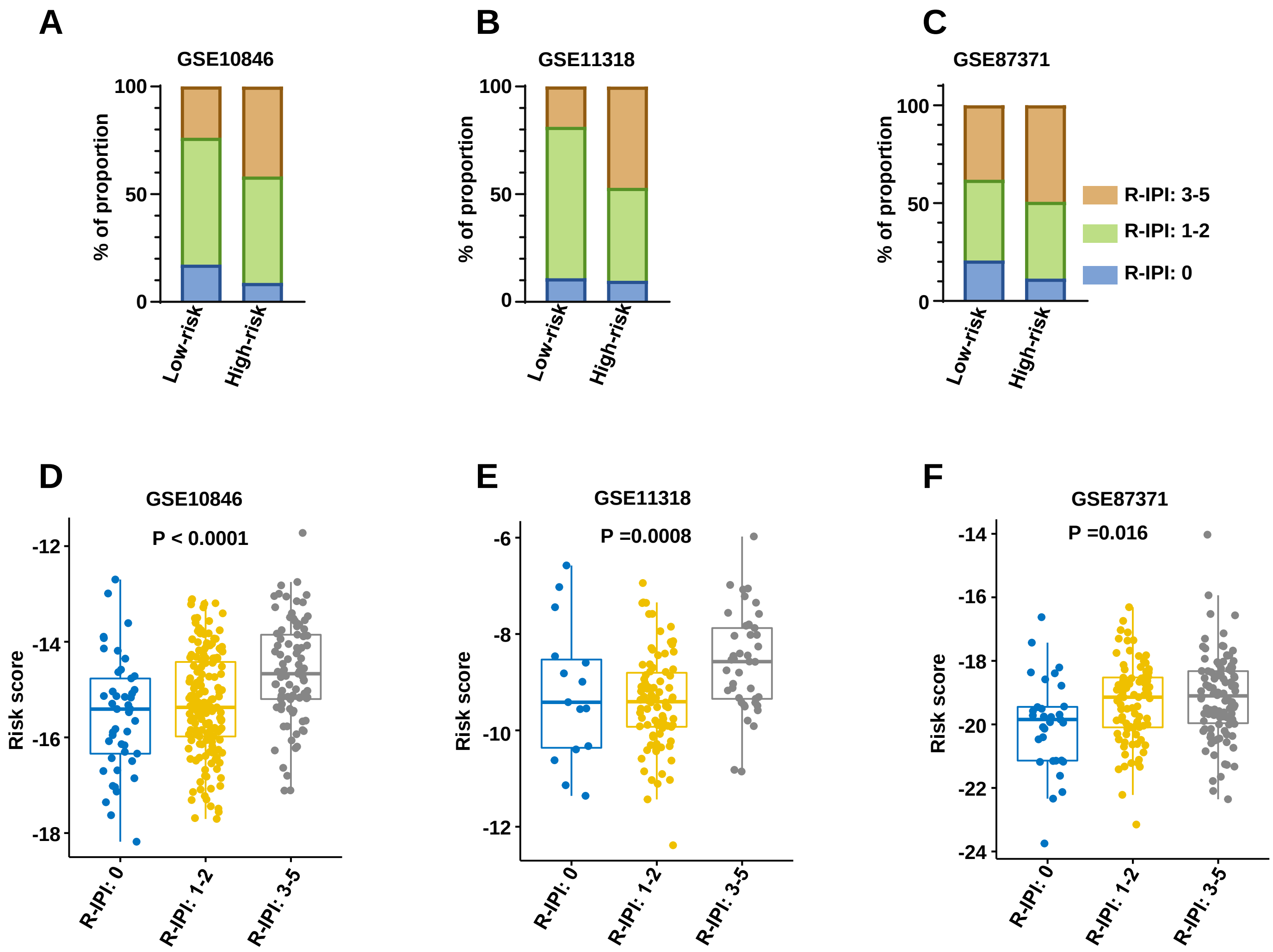
A
GSE10846
% of proportion
100
50
0
Low-risk
High-risk
B
GSE11318
C
GSE87371
R-IPI: 3-5
R-IPI: 1-2
R-IPI: 0
D
GSE10846
P < 0.0001
Risk score
-12
-14
-16
-18
R-IPI: 0
R-IPI: 1-2
R-IPI: 3-5
E
GSE11318
P =0.0008
-6
-8
-10
-12
F
GSE87371
P =0.016
-14
-16
-18
-20
-22
-24

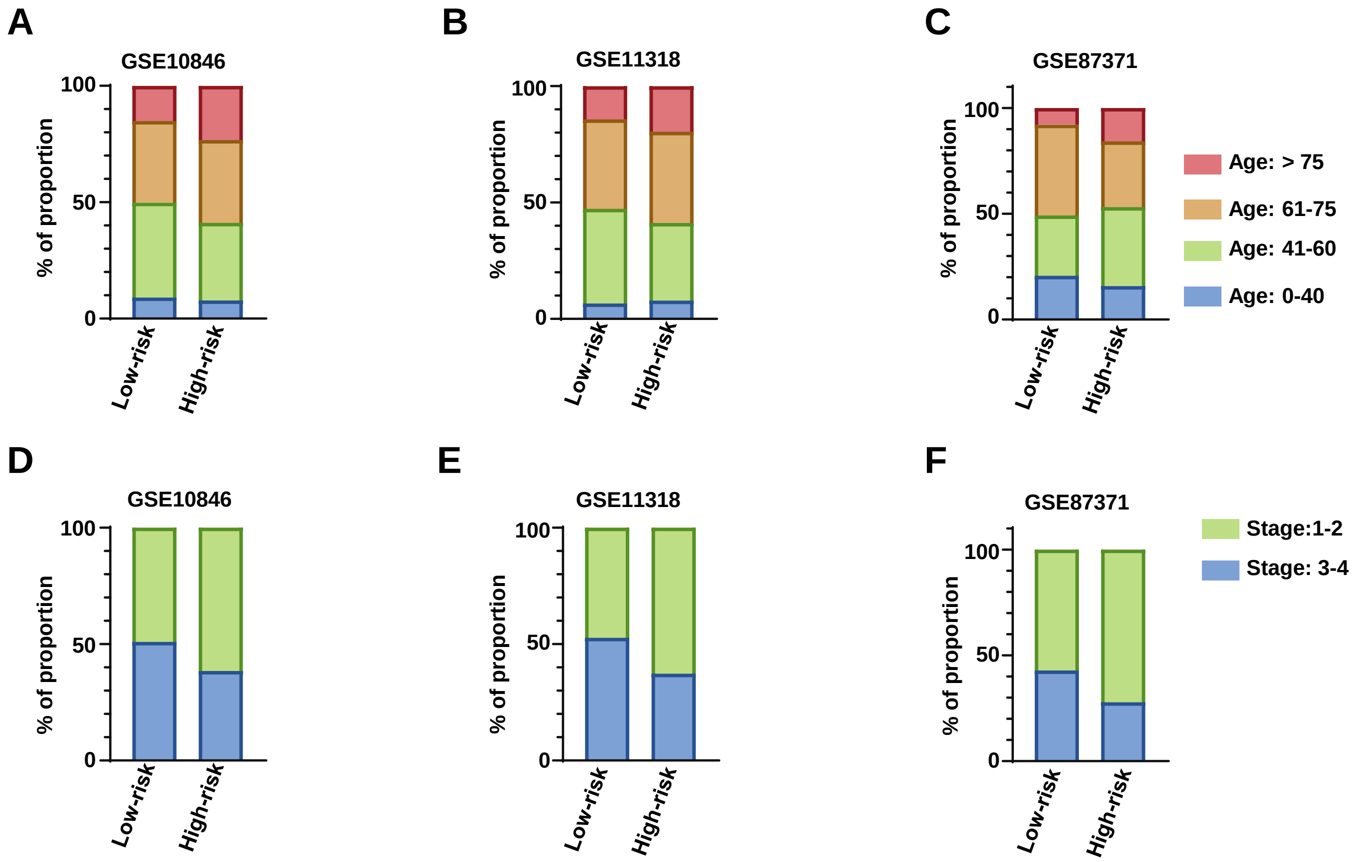

A
GSE10846
B
GSE11318
C
GSE87371
% of proportion
100
50
0
Low-risk
High-risk
Age: > 75
Age: 61-75
Age: 41-60
Age: 0-40
D
GSE10846
E
GSE11318
F
GSE87371
Stage:1-2
Stage: 3-4

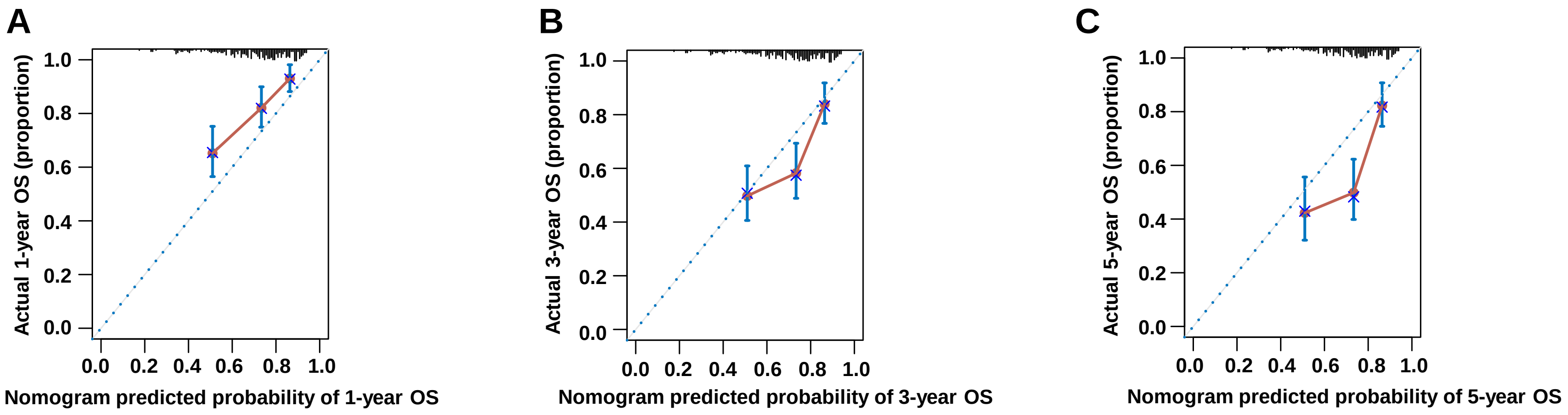

A
B
C
Actual 1-year OS (proportion)
Actual 3-year OS (proportion)
Actual 5-year OS (proportion)
0.0
0.2
0.4
0.6
0.8
1.0
Nomogram predicted probability of 1-year OS
Nomogram predicted probability of 3-year OS
Nomogram predicted probability of 5-year OS

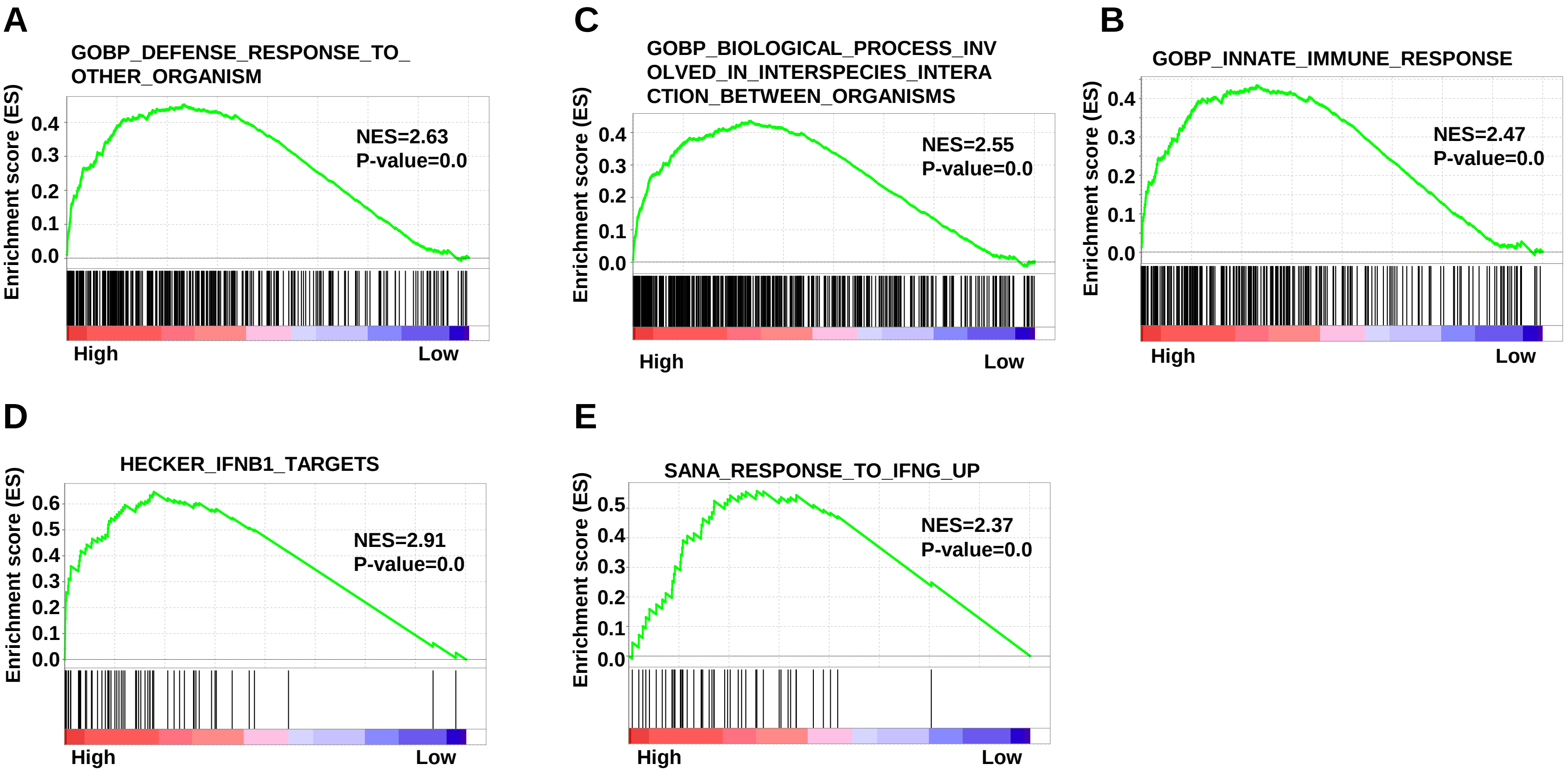
A
GOBP_DEFENSE_RESPONSE_TO_
OTHER_ORGANISM
Enrichment score (ES)
0.4
0.3
0.2
0.1
0.0
NES=2.63
P-value=0.0
High
Low
C
GOBP_BIOLOGICAL_PROCESS_INV
OLVED_IN_INTERSPECIES_INTERA
CTION_BETWEEN_ORGANISMS
Enrichment score (ES)
0.4
0.3
0.2
0.1
0.0
NES=2.55
P-value=0.0
High
Low
B
GOBP_INNATE_IMMUNE_RESPONSE
Enrichment score (ES)
0.4
0.3
0.2
0.1
0.0
NES=2.47
P-value=0.0
High
Low
D
HECKER_IFNB1_TARGETS
Enrichment score (ES)
0.6
0.5
0.4
0.3
0.2
0.1
0.0
NES=2.91
P-value=0.0
High
Low
E
SANA_RESPONSE_TO_IFNG_UP
Enrichment score (ES)
0.5
0.4
0.3
0.2
0.1
0.0
NES=2.37
P-value=0.0
High
Low
